# *Chlamydia suis* displays high transformation capacity with complete cloning vector integration into the chromosomal *rrn-nqrF* plasticity zone

**DOI:** 10.1128/spectrum.02378-23

**Published:** 2023-10-26

**Authors:** Hanna Marti, Michael Biggel, Kensuke Shima, Delia Onorini, Jan Rupp, Steve J. Charette, Nicole Borel

**Affiliations:** 1 Institute of Veterinary Pathology, University of Zurich, Zurich, Switzerland; 2 Institute for Food Safety and Hygiene, University of Zurich, Zurich, Switzerland; 3 Department of Infectious Diseases and Microbiology, University of Lübeck, Lübeck, Germany; 4 Department of Biochemistry, Microbiology and Bioinformatics, Université Laval, Quebec City, Canada; 5 Institut de Biologie Intégrative et des Systèmes, Université Laval, Quebec City, Canada; 6 Centre de Recherche de l’Institut Universitaire de Cardiologie et de Pneumologie de Québec, Quebec City, Canada; LSU Health New Orleans, New Orleans, Louisiana, USA

**Keywords:** *Chlamydiaceae*, *tet*, Tet-island, calcium chloride, native plasmid, transformability, transformation efficiency, lateral gene transfer

## Abstract

*Chlamydia*, comprising several human and zoonotic pathogens, is a genus of the conserved bacterial phylum Chlamydiota. Their obligate intracellular niche serves as a barrier for natural genetic exchange via horizontal gene transfer (HGT), and further limits the development and application of genetic tools. To date, the only example for recent inter-phylum HGT among the Chlamydiota is tetracycline resistance in the potentially zoonotic species *Chlamydia suis*, a close phylogenetic relative of human *C. trachomatis*, which causes bacterial sexually transmitted infections and ocular trachoma. Tetracycline resistance in porcine *C. suis* strains has been described worldwide and is always part of a genomic island dividing *invasin* (*inv*), located within a chromosomal region between the rRNA operon (*rrn*) and the *nqrF* reductase. Here, we aimed to expand the still modest number of available genetic manipulation systems for *Chlamydia* by generating allele-replacement and integration vectors for *C. suis*. These vectors comprised homologous *C. suis* sequences of the chromosomal region of interest, an *E. coli* origin of replication (*ori*) and selection markers but lacked the native chlamydial plasmids or its *ori*. We first recovered allele-replacement mutants using a vector that targets the tryptophan (*trp*) operon of *C. suis*. The vector was further successfully maintained as a free plasmid in *C. trachomatis* without allele replacement, suggesting complex plasmid dynamics in the absence of a chlamydial *ori*. Moreover, we showed that the hypervariable *rrn-nqrF* intergenic region of *C. suis* is highly susceptible to transformation, resulting in complete vector integration upstream of *nqrF* without interruption of the targeted *inv* gene.

**IMPORTANCE:**

The obligate intracellular *Chlamydia* genus contains many pathogens with a negative impact on global health and economy. Despite recent progress, there is still a lack of genetic tools limiting our understanding of these complex bacteria. This study provides new insights into genetic manipulation of *Chlamydia* with the opportunistic porcine pathogen *Chlamydia suis*, the only chlamydial species naturally harboring an antibiotic resistance gene, originally obtained by horizontal gene transfer. *C. suis* is transmissible to humans, posing a potential public health concern. We report that *C. suis* can take up vectors that lack the native plasmid, a requirement for most chlamydial transformation systems described to date. Additionally, we show that *C. trachomatis*, the most common cause for bacterial sexually transmitted infections and infectious blindness worldwide, can be transformed with *C. suis* vectors. Finally, the chromosomal region that harbors the resistance gene of *C. suis* is highly susceptible to complete vector integration.

## INTRODUCTION

The bacterial genus *Chlamydia* harbors almost all clinically relevant chlamydial species of the Chlamydiota phylum, encompassing human, zoonotic, and veterinary pathogens (1). While horizontal gene transfer (HGT) has been described for these obligate intracellular bacteria, it is mostly restricted to intergenus and interspecies transfers (2, 3). Consequently, the only known example of recent interphylum HGT for *Chlamydia* is a distinct tetracycline resistance-conferring genomic island (Tet-island) that has been detected in numerous strains of *Chlamydia suis* worldwide (3). *C. suis* is an opportunistic pathogen primarily located in the gut of pigs and is one of the closest phylogenetic relatives of *C. trachomatis* (4, 5), a causative agent of severe ocular disease and the most common bacterial agent of sexually transmitted infections (STIs) in humans (6). The Tet-island of *C. suis* shares high similarity with a mobilizable plasmid family termed pRAS3 (7, 8), such as pRAS3-3432, recently isolated from *Aeromonas salmonicida* subsp. *salmonicida* (9) and a near-identical plasmid found in *Edwardsiella tarda* (10), both known as bacterial fish pathogens. In *C. suis*, the Tet-island consistently divides the chromosomal *invasin* (*inv*) gene at an identical site (5). This gene is part of a plasticity zone between the rRNA (*rrn*) operon and Na(+)-translocating NADH-quinone reductase subunit F gene (*nqrF*). This *rrn-nqrF* intergenic region is highly diverse within and between *Chlamydia* species (11). While the Tet-island is not present as an extrachromosomal plasmid in *C. suis* (5), the majority of tetracycline-sensitive and tetracycline-resistant *C. suis* strains possess a native plasmid, similar to most *Chlamydia* species (12, 13).

Despite the clinical importance of *Chlamydia*, genetic manipulation tools to achieve a better understanding of chlamydial pathogenesis have only recently expanded (14, 15). To date, the genetic toolbox for *Chlamydia* ranges from chemical mutagenesis to gene disruption by fluorescence-reported allelic exchange mutagenesis (FRAEM) (16) or TargeTron (17), and was recently enhanced by the implementation of CRISPRi technology (18). However, most systems have been implemented using *C. trachomatis* L2, a laboratory strain used to study *Chlamydia* biology, with few exceptions. One of these exceptions includes calcium chloride (CaCl_2_)-mediated transformation with species-specific shuttle vectors that contain at least parts of the native chlamydial plasmid (19 – 23). Many challenges remain such as the extremely low transformation efficiency of *Chlamydia* in general (24) or the complete lack of transformation systems for nearly all veterinary chlamydial pathogens, for example, *C. pecorum*, *C. abortus*, or *C. suis*, though many of these species could be detrimental to animal and human health (1).

We hypothesized that *C. suis* could be a highly transformable species due to its natural capacity for HGT. Our first aim was to establish a transformation system for *C. suis* using species-specific shuttle vectors. Furthermore, we aimed to expand the genetic toolbox of *Chlamydia* by developing an allele-replacement mutagenesis system that lacks the native *C. suis* plasmid or its origin of replication (*ori*). Finally, we used similar vectors to investigate the *rrn-nqrF* intergenic region as a potential target for HGT. We successfully established a CaCl_2_-mediated transformation protocol for *C. suis* and optimized the protocol by comparing the number of transformants at the third passage between different conditions. We developed an allele-replacement mutagenesis protocol for *C. suis* targeting the *trp* region. Additionally, we show that *C. trachomatis* can be transformed with this *C. suis* vector, though no allelic exchange was observed. Finally, we demonstrate that *C. suis* is highly susceptible to transformation and integration in the intergenic *rrn-nqrF* plasticity zone. Specifically, using several vectors based on *inv*, we consistently achieved complete vector integration upstream of *nqrF* without disrupting the original *inv* gene.

## RESULTS

### *C. suis* transformation is more efficient at 100–200 mM compared to 50 mM CaCl_2_

We established the first CaCl_2_-mediated transformation protocol for *C. suis* using a shuttle vector termed pUC-Cspl-GFP for which we replaced the *C. trachomatis* plasmid sequence of pGFP::SW2 (19) with that of the *C. suis* laboratory strain S45 (Table 1). We then optimized our initial *C. suis* transformation protocol (19, 21) by comparing various conditions such as CaCl_2_ concentration, time of initial selection, the choice of antibiotic selection, the method of cell preparation prior infection, and the strain used for transformation. In contrast to many extracellular bacteria (25), there is no published protocol to determine transformation efficiency in *Chlamydia*. In this study, we compared the transformation success between different growth conditions by determining the number of transformed inclusions at the third passage (Passage 3), expressed as inclusion forming units per mL (IFU/mL).

**TABLE 1:** List of chlamydial strains used in this study

Species	Strain (alt. name)	Reference	Accession no.
*C. suis*	S45 RIF, derived from S45/6	(12, 26, 27)	CP063064
*C. suis*	SWA-94 (10–26b)	(5, 28)	PRJEB17986
*C. suis*	SWA-141 (4–29b)	(5, 28)	PRJEB17986
*C. trachomatis*	Plasmid-free L2	(29)	Provided by the Chlamydia Biobank^a^
*C. muridarum*	Weiss	(30, 31)	Provided by Kyle Ramsey, Midwestern University, IL, USA

^a^ https://www.chlamydiabiobank.co.uk/.

Optimization experiments revealed that CaCl_2_ concentrations ranging from 100 to 200 mM led to significantly more transformants compared to 50 mM (Fig. S1A). We obtained the same number of transformants if ampicillin was added at 6-, 12-, or 24-hr post-infection (hpi), or not, prior to the first passage (Fig. S1B). Moreover, more transformants were obtained following selection in ampicillin compared to chloramphenicol (Fig. S1C) and there were 1,000-fold more transformants if initial infection post CaCl_2_ treatment was performed in trypsinized cells (Tryps) as opposed to a confluent monolayer (Seed, Fig. S1D). Finally, the laboratory strain S45 RIF yielded more transformants than the field strain SWA-94 in three independent experiments (Fig. S1E). Taken together, the *C. suis* transformation protocols used in this study included the use of 100 mM CaCl_2_ for a 1-hr incubation of the vector and *C. suis* EBs at room temperature, followed by infection of trypsinized cells prior to centrifugation. Initial selection was then performed with ampicillin at 6 hpi, using the laboratory strain S45 RIF.

### A *C. suis trp* allele-replacement vector lacking the chlamydial *ori* was transformed into *C. suis* and *C. trachomatis,* with allelic exchange occurring only in *C. suis*

**Fig 1:**
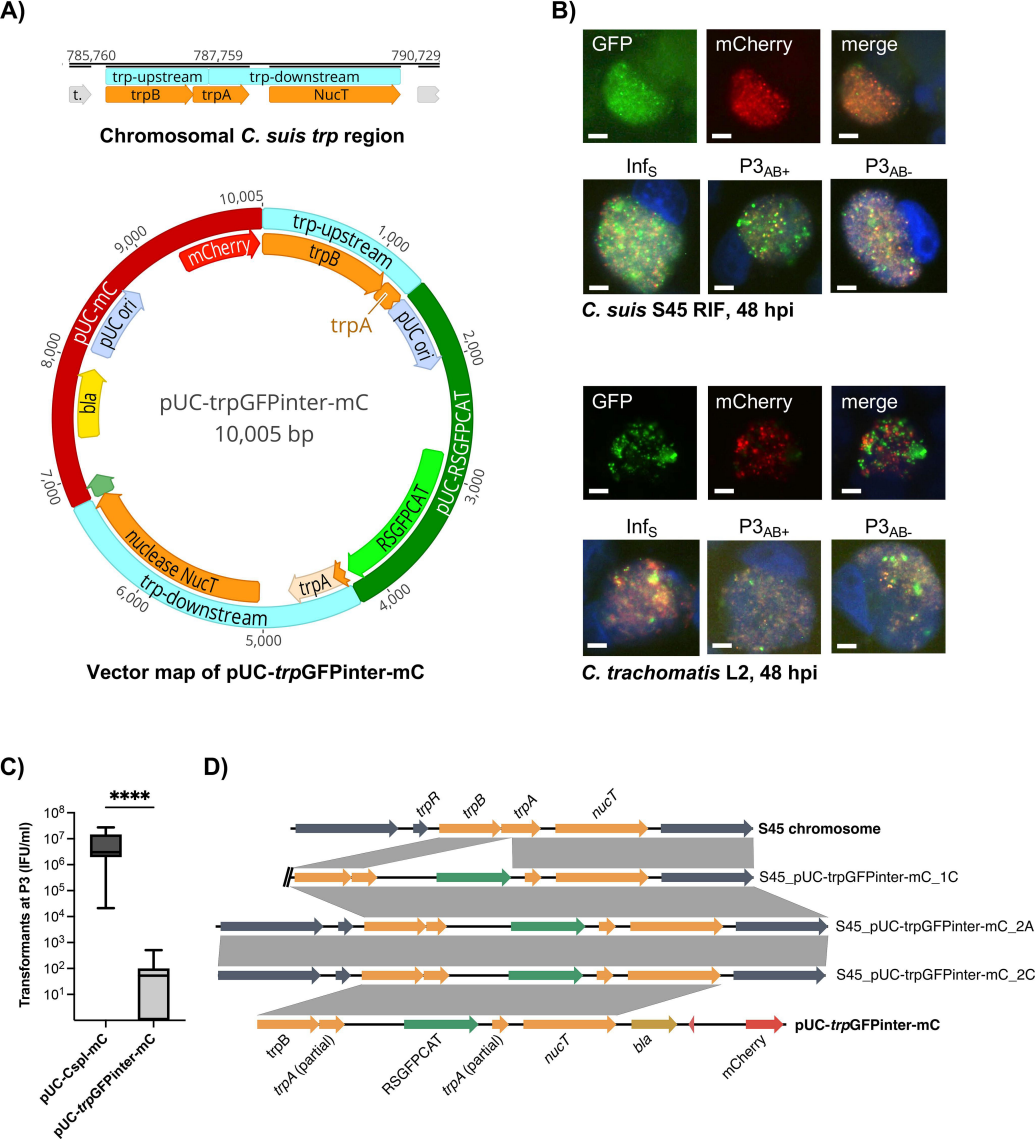
A *C. suis trp* allele-replacement vector lacking the chlamydial *ori* was transformed into *C. suis* and *C. trachomatis,* with allelic exchange occurring only in *C. suis*. (**A**) Shown is the map of the chromosomal *trp* region of *C. suis* and the *trp* allele-exchange vector pUC-*trp*GFPinter-mC. The *trp* region (*trpB, trpA*, nuclease NucT) is indicated in orange, the interruption is indicated by two blue boxes, specifically *trp* upstream (1,379 bp, *trpB* and partial *trpA*) and *trp* downstream (2,575 bp, partial *trpA* and NucT). The backbone vector pUC-mC (dark red) derives from pBOMB4R-MCI (33) and consists of a promoter (green), a beta-lactamase (*bla*, yellow), a pUC origin of replication (pUC *ori*, pale blue), and mCherry (red). The *trp* region is interrupted by pUC-RSGFPCAT (dark green), which is derived from pGFP::SW2 (19) and comprises a pUC *ori* and the fusion gene RSGFPCAT (green) containing a red-shifted GFP gene and a chloramphenicol resistance gene (*cat*), and its associated promoter (not shown). (**B**) Shown are representative images of *C. suis* (top panel) and *C. trachomatis* (bottom panel) cultures successfully transformed with pUC-*trp*GFPinter-mC in ampicillin. The top rows of each panel show the inclusion of a transformant at 48-hr post-infection (hpi), positive for RSGFPCAT (green fluorescence, left) and mCherry (red fluorescence, middle). Images were taken individually and then merged (right). The bottom rows display representative images of the stability assay for each transformant, following infection in ampicillin (Inf_S_) and after three passages in the presence (P3_AB+_) and absence (P3_AB-_) of ampicillin. DAPI staining (blue) visualized DNA and was merged with images taken in the green and red channels. Size bar indicates 5 µm. (**C**) Transformant progeny was determined by counting the number of transformants at Passage 3, expressed as inclusion forming units per mL (IFU/mL). We compared the number of *C. suis* plasmid shuttle vector transformants (pUC-Cspl-mC) with that of *trp* allele-exchange vector pUC-trpGFPinter-mC mutants following three independent experiments. Four asterisks (****) represent *p* values <0.0001. (**D**) Sequence comparison of vector pUC-*trp*GFPinter-mC, the chromosomal *trpRBA* region in native strain S45, and the corresponding region in three transformants. Gray boxes between sequences indicate homologous regions, orange arrows indicate homologous ORFs of the S45 chromosome and pUC-*trp*GFPinter-mC. Chromosomal regions are shown in dark blue; RSGFPCAT, *bla*, and mCherry are shown in green, dark yellow, and red, respectively. The figure was generated using EasyFig 2.1 (34).

We then challenged the hypothesis that vectors require the presence of the entire chlamydial plasmid (19), or at least its origin of replication (*ori*) (23), for stable transformation into *Chlamydia*. We constructed an allele-replacement vector targeting the *trpBA* operon of *C. suis* (Fig. 1A). This region is involved in tryptophan synthesis and was successfully used as proof of principle for *C. trachomatis* transformation in a previous study (16). The resulting vector pUC-*trp*GFPinter-mC (Fig. 1A) was 10 kilobase pairs (kbp) in size. Its *trp* operon was interrupted by a pUC *ori* and the RSGFPCAT cassette of pGFP::SW2 (19), consisting of a fusion gene encoding a red-shifted GFP (RSGFP) and a chloramphenicol resistance gene (*cat*). The backbone vector included a beta-lactamase (*bla*), pUC *ori*, and mCherry. We attempted to transform this vector into *C. suis*, *C. trachomatis*, and *C. muridarum*, the *Chlamydia* species used in urogenital mouse models (32). Species-specific shuttle vectors were used in parallel and served as transformation controls. For *C. suis*, we replaced the *C. trachomatis* plasmid structure of pBOMB4R-MCI with that of *C. suis*, resulting in the 10.7 kbp vector pUC-Cspl-mC (Table 2). For *C. trachomatis* and *C. muridarum*, we used pBOMB4R-MCI and pUC-Cmpl-mC, respectively (Table 2).

**TABLE 2:** Overview vectors used in this study

Name	Size (bp)	Resistance	Marker	*C. suis (Cs)/Chlamydia* insert
pUC-mC	3,214	*bla*	mC	None
pUC-Cspl-GFP	11,852	*bla, cat*	GFP	*C. suis* plasmid
pUC-Cspl-mC	10,708	*bla*	mC	*C. suis* plasmid
pUC-*inv*up-GFP	4,217	*cat*	GFP	*Cs inv* upstream of Tet-island (1,380 bp)
pUC-*inv*up-mC	4,594	*bla*	mC	*Cs inv* upstream of Tet-island (1,380 bp)
pUC-*inv*down-mC	5,832	*bla*	mC	*Cs inv* downstream of Tet-island (2,618 bp)
pUC-*inv*GFPinter-mC	10,049	*bla, cat*	mC, GFP	*Cs inv* interrupted by RSGFPCAT
pUC-*inv*com-mC	7,212	*bla*	mC	*Cs inv* complete
pUC-*inv*GFPinter500-mC	7,051	*bla, cat*	mC, GFP	*Cs inv* interrupted by RSGFPCAT, 500 bp up/down
pUC-*trp*up-GFP	4,216	*cat*	GFP	*Cs trpB* (1,379 bp)
pUC-*trp*down-mC	5,789	*bla*	mC	*Cs trpA*/NucT (2,575 bp)
pUC-*trp*GFPinter-mC	10,005	*bla, cat*	mC, GFP	*Cs trpB* / *trpA-NucT* interrupted by RSGFPCAT
pUC-*inv*pRAS3inter-mC	13,403	*bla, tet*	mC	*Cs inv* interrupted by pRAS3-3432 (6,169 bp)
pUC-Cspl-GFPddbla	10,331	*cat*	GFP	*C. suis* plasmid
pUC-pRAS3-Cspl-GFPCAT	16,522	*cat, tet*	GFP	*C. suis* plasmid
pUC-Cmpl-mC	10,175	*bla*	mC	*C. muridarum* plasmid
pBOMB4R-MCI	10,768	*bla*	mC	*C. trachomatis* plasmid

Transformation with pUC-*trp*GFPinter-mC was successful for both *C. suis* and *C. trachomatis* in at least one independent experiment (Fig. 1B). Transformation of pUC-*trp*GFPinter-mC into *C. muridarum* was not successful in three independent experiments (Fig. S2A). *C. suis* control vector pUC-Cspl-mC yielded significantly more transformants compared to pUC-*trp*GFPinter-mC (Fig. 1C). The *C. trachomatis* control vector pBOMB4R-MCI could not be transformed with the transformation protocol used in this study (data not shown).

Suspected transformant cultures were collected at the fourth passage and then passaged another six times in the presence of either ampicillin (25 µg/mL) or chloramphenicol (1 µg/mL) for the generation of stocks in sucrose phosphate glutamate buffer. The fluorescent signal observed in the inclusions of suspected transformants was then maintained for an additional three passages in the presence and absence of ampicillin (Fig. 1B) or chloramphenicol (Figure S2B through C). We observed a continuous, but not complete, loss of fluorescent signal in the inclusions of *C. trachomatis* transformants over 13 performed passages while the fluorescent signal remained consistent in the inclusions of all *C. suis* transformants. Furthermore, we observed that, while inclusions of *C. trachomatis* transformants were always positive for both GFP and mCherry (Fig. 1B), both double-fluorescent and GFP-only *C. suis* inclusions were present (Fig. S2B). The signal of GFP-only inclusions was weaker and homogeneously distributed compared to the heterogeneous and intense GFP signal in double-fluorescent inclusions. Overall, these GFP-only inclusions are indicative for allele replacement with the *C. suis trp* region, similar to what was described for the FRAEM method (16). Consequently, we expected stable transformation for both *C. trachomatis* and *C. suis* but allele replacement only for *C. suis*.

For confirmation of allele replacement, we performed long-read sequencing of two suspected *C. suis* mutants from two separate experiments, with either ampicillin or chloramphenicol used for stock preparation resulting in four separate sequencing reactions (Table S1). As suspected, genome assemblies generated with Flye showed that *trpA* was interrupted by the RSGFPCAT cassette, confirming allele replacement (Fig. 1D). The *bla-*mCherry backbone was identified in all four transformants, accounting for the red fluorescent signal detected in the cultures. DNA of the allele-replacement mutants was then used for transformation into highly competent *Escherichia coli* to confirm the circularity of the vectors. Free plasmid could be obtained from all samples, though vectors from allele-replacement mutants grown in ampicillin lacked the RSGFPCAT cassette (Table S1). The native plasmid was identified in all four genome assemblies, whereas transformants with control vector pUC-Cspl-mC lacked the native plasmid (Table S1).

In contrast to these findings, pUC-*trp*GFPinter-mC was only detected as a circular plasmid in *C. trachomatis* without any sign of integration into the chromosome. In the transformant grown in ampicillin, the vector was present as a dimer (Table S1). We suspect that the low homology (79%) between the plasmid *trp* region derived from *C. suis* and the chromosomal *C. trachomatis trp* region prevented homologous recombination and therefore allele replacement.

### CaCl_2_-mediated transformation of an *inv* allele-replacement vector leads to integration and amplification without disruption of chromosomal *inv* in *C. suis*

**Fig 2:**
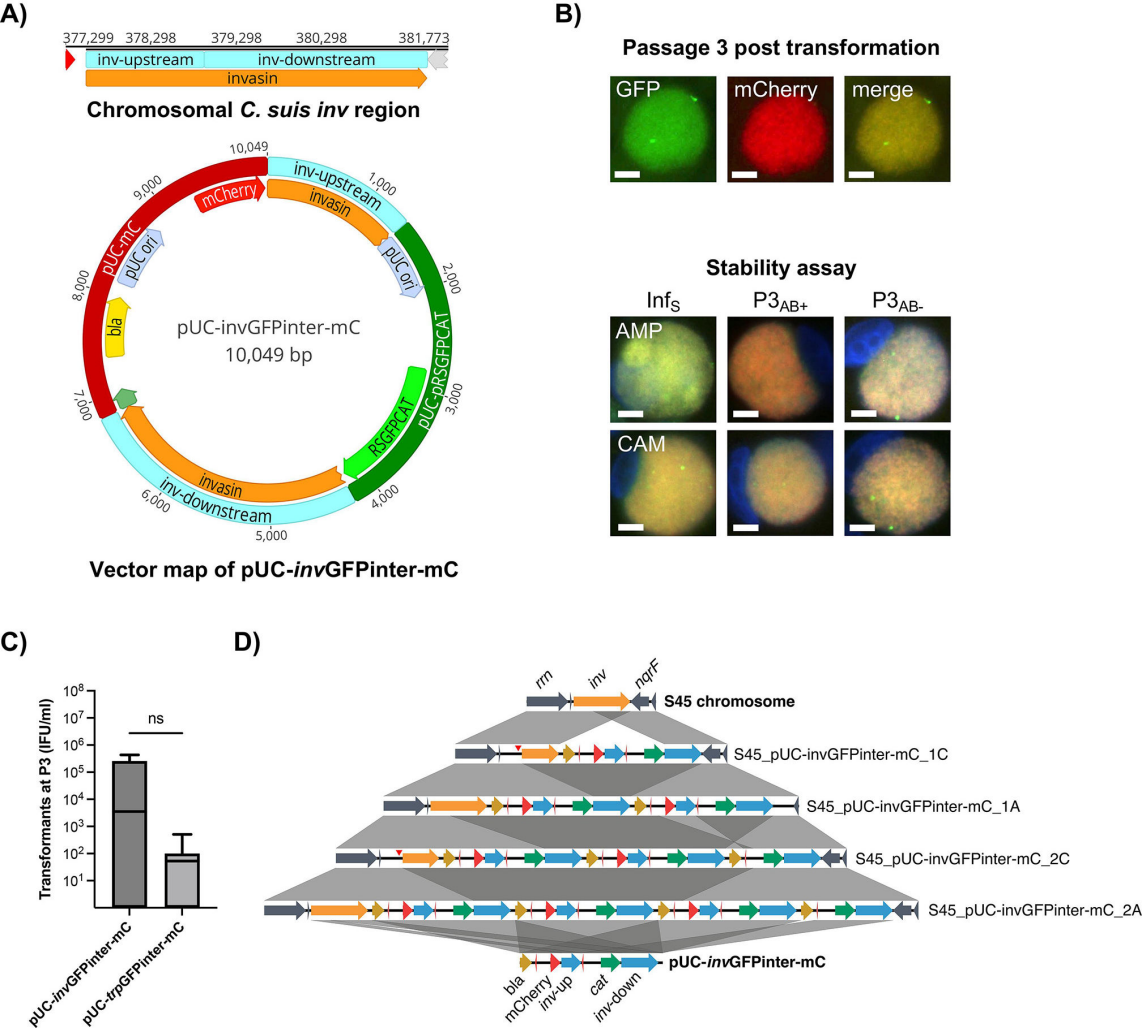
CaCl_2_-mediated transformation of an *inv* allele-replacement vector into *C. suis* leads to complete vector integration without interruption of chromosomal *inv*. (**A**) Shown is the map of the chromosomal *inv* region of *C. suis* and the resulting interrupted *inv* vector pUC-*inv*GFPinter-mC. The *inv* region is indicated in orange, the interruption is indicated by two blue boxes, specifically *inv* upstream (1,380 bp) and *inv* downstream (2,613 bp), and is identical to the insertion site of the Tet-island (7). The backbone vector pUC-mC (dark red) derives from pBOMB4R-MCI (33) and consists of a promoter (green), a beta-lactamase (*bla*, yellow), a pUC *ori* (pale blue), and mCherry (red). The *inv* region is interrupted by pUC-RSGFPCAT (dark green), which is derived from pGFP::SW2 (19) and comprises a pUC *ori* as well as the fusion gene RSGFPCAT (green) consisting of a red-shifted GFP gene and a chloramphenicol resistance gene (*cat*), and its associated promoter. (**B**) Shown are representative images of *C. suis* strain S45 RIF successfully transformed with pUC-*inv*GFPinter-mC. The top row shows the representative inclusion of a transformant at 48-hr post-infection, positive for RSGFPCAT (green fluorescence, left) and mCherry (red fluorescence, middle). Images were taken individually and then merged (right). The bottom row shows representative images of the stability assay for each transformant, following infection in ampicillin (Inf_S_) and after three passages in the presence (P3_AB+_) and absence (P3_AB−_) of ampicillin (AMP, top row) or chloramphenicol (CAM, bottom row). DAPI staining (blue) visualizes DNA and was merged with images taken in the green and red channels. Size bar indicates 5 µm. (**C**) Transformant progeny was determined by counting the number of transformants at Passage 3, expressed as inclusion forming units per mL (IFU/mL). We compared the number of *trp* allele-replacement vector pUC-*trp*GFPinter-mC mutants with that of *invasin* vector pUC-*inv*GFPinter-mC transformants following three independent experiments. Non-significant results are indicated with ns. (**D**) Sequence comparison of vector pUC-*inv*GFPinter-mC, the chromosomal *inv* region in native strain S45 (orange), and the corresponding region in four transformants. Gray boxes between sequences indicate homologous regions, blue arrows indicate homologous *inv*-up and *inv*-down ORFs of pUC-invGFPinter-mC. Additional chromosomal regions are shown in dark blue; *bla*, mCherry, and *cat* are shown in dark yellow, red, and green, respectively. Deletions leading to contraction of the ORFs are highlighted with a vertical arrow tip (red). The figure was generated using EasyFig 2.1 (34).

Next, we targeted the *rrn-nqrF* plasticity zone of *C. suis* by constructing an allele-replacement vector targeting *inv,* using the same backbone structure and homology arm lengths as for the *trp* vector (Fig. 2A). While transformation with pUC-*inv*GFPinter-mC was not successful for *C. trachomatis* or *C. muridarum* (Fig. S3A), we obtained transformants with double-fluorescent inclusions for *C. suis* strain S45 RIF. The fluorescent signal was more homogeneously distributed than that of *trp* transformants and more intense than expected (Fig. 2B). The fluorescent signal for GFP and mCherry remained consistent in all transformants over 13 passages including three passages in the absence of selective antibiotics (Fig. 2B).

Similar to *trp* allele-replacement mutants, stock preparation and stability testing were performed both in chloramphenicol and ampicillin (Fig. 2B). We then compared the number of transformed inclusions at Passage 3 between *C. suis* transformed with the *trp* and *inv* vectors. Although there was no significant difference between the two vectors (Fig. 2C), average transformant progeny was 1.4 × 10^2^ and 2 × 10^5^ IFU/mL for pUC-*trp*GFPinter-mC and pUC-*inv*GFPinter-mC, respectively.

Based on the homogenous, double-fluorescent signal in transformed inclusions, we hypothesized that pUC-*inv*GFPinter-mC is maintained as a circular plasmid in multiple copies, with limited or no allele replacement. However, sequencing of two separate transformant cultures, grown in both ampicillin and chloramphenicol (*n* = 4), revealed complete cloning vector integration into the chromosome. Subsequent amplification of the integrants was observed in all but one transformant (Fig. 2D; Table S1). The site of integration was consistently located downstream of chromosomal *inv*, leaving the gene intact. This integration resulted in the expansion of the chlamydial chromosome by at least 10 kbp upstream of *nqrF* and therefore within the *rrn-nqrF* plasticity zone (Fig. 2D). Additionally, both the native plasmid and a circular pUC-*inv*GFPinter-mC was maintained in the cultures regardless of antibiotic selection, the latter of which was confirmed by the transformation of pUC-*inv*GFPinter-mC into highly competent *E. coli* (Table S1).

Finally, we attempted transformation of field strain SWA-141 (Table 1), a strain containing the complete 12 kbp Tet-island, thus dividing *inv* at the same site as pUC-*inv*GFPinter-mC. No transformants were obtained from three independent transformation experiments (Fig. S3B). Using control vector pUC-Cspl-mC, we determined the average number of SWA-141 transformants at the third passage and found that it was 10-fold lower than that of S45 RIF (Fig. S2B).

### Homology arms of 1 kb or greater allow successful integration of *inv* vectors into the *rrn-nqrF* plasticity zone

**Fig 3:**
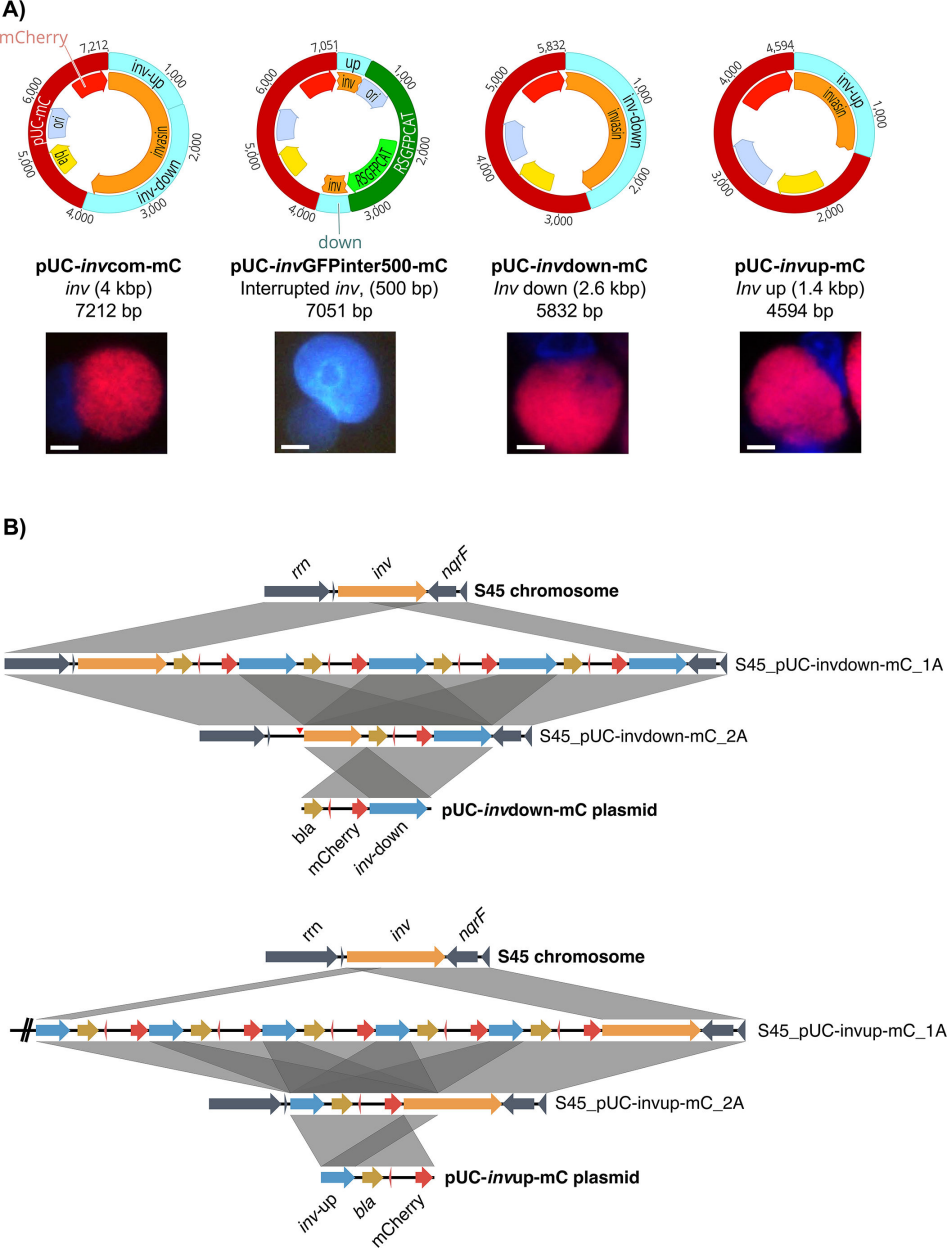
Homology arms of 1 kb or greater allow successful integration of *inv* vectors into the *rrn-nqrF* plasticity zone. (**A**) Shown are the maps of all additional vectors related to the *inv* region, namely the complete, uninterrupted *inv* vector pUC-*inv*com-mC, a truncated *inv* vector pUC-*inv*GFPinter500-mC, as well as vectors containing the downstream (pUC-*inv*down-mC) and upstream homology arm (pUC-*inv*up-mC) of pUC-*inv*GFPinter-mC. The *inv* region is indicated in orange, the interruption is indicated by two blue boxes, specifically *inv* downstream (2,613 bp) and upstream (1,380 bp). The backbone vector pUC-mC (dark red) derives from pBOMB4R-MCI (33) and consists of a promoter (green), a beta-lactamase (*bla*, yellow), a pUC origin of replication (pUC *ori*, pale blue), and mCherry (red). For pUC-*inv*GFPinter500-mC, the *inv* region is interrupted by pUC-RSGFPCAT (dark green), which is derived from pGFP::SW2 (19) and comprises a pUC *ori* and the fusion gene RSGFPCAT (green) containing of a red-shifted GFP gene and a chloramphenicol resistance gene (*cat*), and its associated promoter. The *inv* sequences are 500 bp long, both up- and downstream. Below each vector, representative images of transformation attempts are shown. Cultures were stained with DAPI (blue) and images were taken for mCherry (red channel) and DAPI. Bars indicate 5 µm. (**B**) The top shows sequence comparison of vector pUC-*inv*down-mC, the chromosomal *inv* region in native strain S45 (orange), and the corresponding region in two transformants, while the bottom shows a similar sequence comparison of plasmid pUC-*inv*up-mC, chromosomal S45, and two transformants. The figures were generated using EasyFig 2.1 (34). Gray boxes between sequences indicate homologous regions, blue arrows indicate homologous *inv*-up and *inv*-down ORFs of pUC-invGFPinter-mC. Additional chromosomal regions are shown in dark blue; *bla* and mCherry are shown in dark yellow and red, respectively. Deletions leading to contraction of the ORFs are highlighted with a vertical arrow tip (red).

We did not expect complete integration of *inv* vector pUC-*inv*GFPinter-mC into the *rrn-nqrF* region without interruption of chromosomal *inv*. These findings taken together with the fact that this region naturally obtained the Tet-island (7) confirm the plasticity of the region as well as the stability of *nqrF*. By creating four modified pUC-*inv*GFPinter-mC vectors (Fig. 3A), we aimed to investigate the integration dynamics of *inv* vectors. First, we removed the RSGFPCAT cassette, resulting in the 7.2 kbp vector pUC-*inv*com-mC with an intact *inv* gene of 4 kbp. Second, we shortened the homology arms to 500 bp both up- and downstream of the RSGFPCAT cassette, generating the 7 kbp vector pUC-*inv*GFPinter500-mC. Finally, we removed the RSGFPCAT cassette together with either the up- or downstream homology arm of the vector, thus generating vectors pUC-*inv*down-mC (5.8 kbp, *inv*: 2.6 kbp) and pUC-*inv*up-mC (4.5 kbp, 1.4 kbp), respectively (Fig. 3A; Table 1). Stable transformation was possible for all vectors except pUC-*inv*GFPinter500-mC (Fig. 3A; Fig. S4A). The number of transformed inclusions at the third passage was not significantly different for pUC-*inv*com-mC (1.6 × 10^5^ to 1.1 × 10^6^ IFU/mL) and pUC-*inv*down-mC (1.1 × 10^2^ to 3.4 × 10^4^ IFU/mL), likely due to interexperimental variation. In contrast, pUC-*inv*up-mC yielded significantly fewer transformants (0–2.7 × 10^1^ IFU/mL) compared to the previous two vectors (Fig. S4B).

For each vector, we sequenced two *C. suis* transformants for further analysis (*n* = 6). In all six cultures, we detected complete vector integration upstream of the *nqrF* gene (Table S1). Similar to the original *inv* vector, integrant amplification was detected in four out of six transformants, without disruption of *inv* (Fig. 3B; Fig. S4C). The exact site of integration depended on the structure of the vector. While for pUC-*inv*down-mC, the site of integration was identical to the original pUC-*inv*GFPinter-mC vector, downstream of *inv* and upstream of *nqrF*, the vector with the upstream *inv* homology arm (pUC-*inv*up-mC) integrated downstream of the *rrn* operon and upstream of *inv* (Fig. 3B). Considering that the *inv* region of pUC-*inv*com-mC was identical to that of the original *inv*, the exact site of integration could not be determined (Fig. S4C). For all transformants, the native plasmid was present and free vector could be re-transformed into competent *E. coli*, without notable changes to the vector sequence (Table S1).

### *Inv* vectors promote integration of up to 10 kbp non-chlamydial DNA into the *rrn-nqrF* region without disruption of the original *inv* gene

Taking into consideration the high transformability of *C. suis* as well as the natural integration of the Tet-island, which is of non-chlamydial origin (7), we investigated the capability of *C. suis* to integrate non-chlamydial DNA into its chromosome. For this analysis, we included vectors from two bacteria: *E. coli* and *A. salmonicida* subsp. *salmonicida*. For *E. coli*, we used pBOMB4R-MCI to construct vector pUC-mC, comprising only the pUC *ori*, *bla*, and mCherry without any chlamydial DNA (Table 1; Fig. S5A). As a *A. salmonicida* subsp. *salmonicida* vector, we used pRAS3-3432, the plasmid with the highest homology to the Tet-island (Table 1; Fig. S5A). We hypothesized that it is possible to transform pRAS3-3432, but not pUC-mC, into *C. suis* (Table 1; Fig. S5A). As a positive transformation control, we used shuttle vector pUC-Cspl-GFP (Table 1; Fig. S5B). Three transformation attempts were performed, aiming for an average of at least 1 × 10^6^ IFU/mL transformant progeny at Passage 3 in the control vector. However, neither pUC-mC nor pRAS3-3432 could be transformed into *C. suis* (Fig. S5A). Given these findings, we concluded that, with the currently described protocol, chlamydial DNA is a crucial component for successful transformation into *C. suis*.

**Fig 4:**
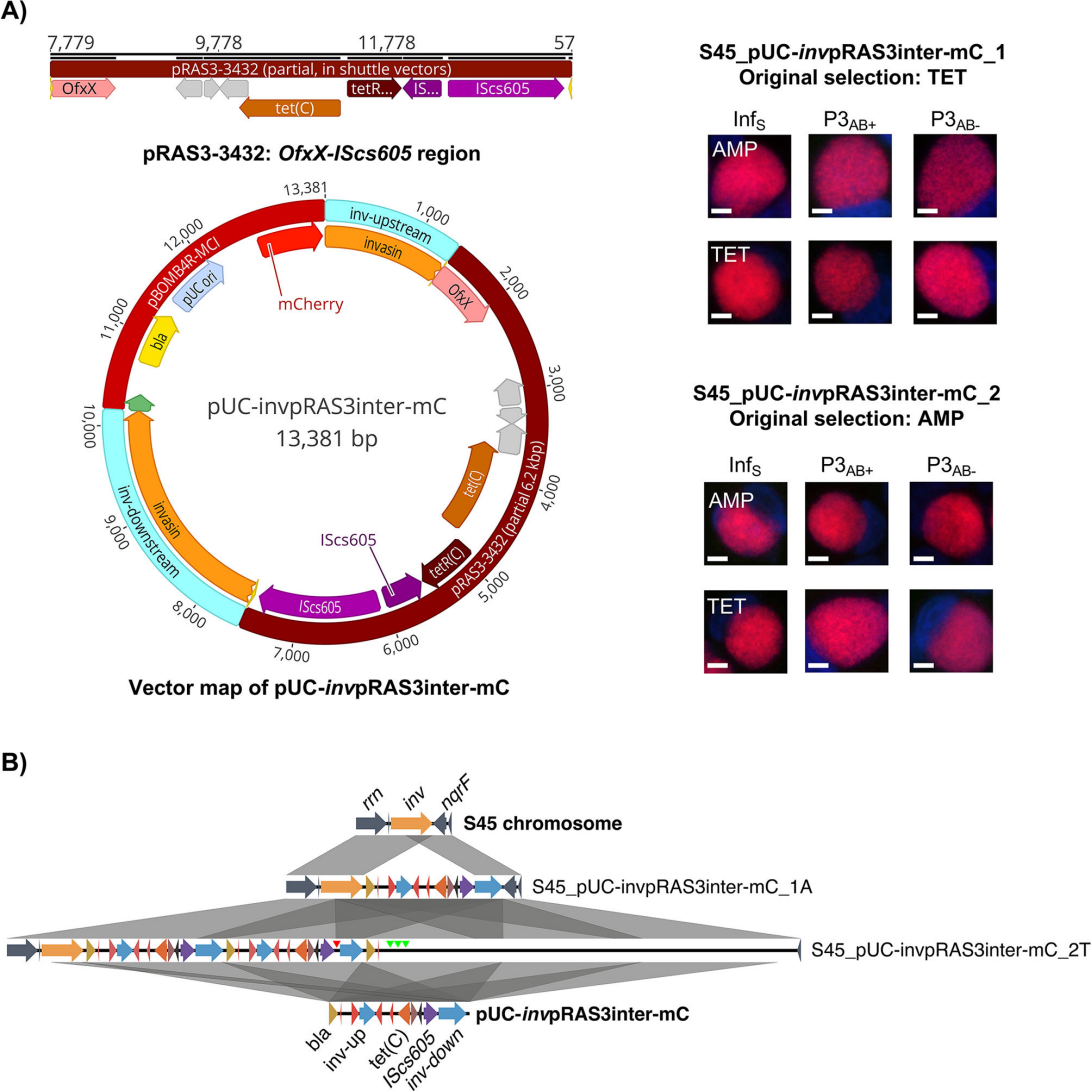
*Inv* vectors promote integration of up to 10 kbp non-chlamydial DNA into the *rrn-nqrF* region without disruption of the original *inv* gene. (**A**) Shown is the *OfxX-IScs605* sequence of pRAS3-3432 used for vector construction (top) and the vector map of pUC-*inv*pRAS3inter-mC (bottom). Moreover, the right panel shows representative images of stable transformants obtained from tetracyline (TET, top) and ampicillin (AMP) selection (bottom). Cultures following infection with AMP (Inf_S_, top) or TET (bottom), and after three passages in the presence (P3_AB+_) and absence (P3_AB-_) of the corresponding selection were fixed and stained with DAPI. Images were taken for the DAPI (blue) and red channel (mCherry), and merged. Bars indicate 5 µm. (**B**) Sequence comparison of vector pUC-*inv*pRAS3inter-mC, the chromosomal *inv* region in native strain S45 (orange), and the corresponding region in two transformants. Gray boxes between sequences indicate homologous regions, blue arrows indicate homologous *inv*-up and *inv*-down ORFs of pUC-invpRAS3inter-mC. Additional chromosomal regions are shown in dark blue; *bla*, *tetA*(C), and *IScs605* are shown in dark yellow, dark orange, and black/purple, respectively. Deletions leading to contraction of the ORFs are highlighted with a vertical arrow tip (red). Multiple deletions leading to mismatch over long sequences are indicated with three vertical green arrow tips. The figure was generated using EasyFig 2.1 (34).

Next, we used a 6.2 kbp sequence of pRAS3-3432, covering the *OfxX-IScs605* region (Fig. 4A), including *tetA*(C)/*tetR*(C) and the *IScs605* transposase, to construct two vectors, pUC-pRAS3-Cspl-GFPCAT (Fig. S5C) and pUC-*inv*pRAS3inter-mC (Fig. 4A). Specifically, *C. suis* vector pUC-pRAS3-Cspl-GFPCAT comprised pUC-RSGFPCAT as *E. coli* backbone, the *C. suis* plasmid and the *OfxX-IScs605* sequence of pRAS3-3432 (16.5 kbp, Table 1; Fig. S5C). For the construction of *C. suis inv* vector pUC-*inv*pRAS3inter-mC, the RSGFPCAT cassette of pUC-*inv*GFPinter-mC was replaced by the *OfxX-IScs605* region of pRAS3-3432 (13.4 kbp, Table 1; Fig. 4A). Transformation of *C. suis* vector pUC-pRAS3-Cspl-GFPCAT was unsuccessful in four independent experiments, using either tetracycline (0.125 µg/mL) or chloramphenicol (0.5 µg/mL) for selection (Fig. S5C). In contrast, the *inv* vector yielded stable transformants following both, selection with tetracycline and ampicillin (Fig. S5D). Whole-genome sequencing revealed complete cloning vector integration upstream of *nqrF* and downstream of the intact *inv* gene in two out of four transformants (Fig. 4B; Table S1). Multiple copies of the integrant were detected in one culture. The vector was additionally present as a circular plasmid without replacement of the native plasmid and could be retrieved from highly competent *E. coli* (Table S1).

## DISCUSSION

Current transformation protocols for *Chlamydia* focus on *C. trachomatis* or *C. muridarum* (14) and either require the use of cloning vectors with a broad host range (35) or the implementation of chlamydial plasmid sequences into the vector of choice, requiring at least the chlamydial *ori* (23).

Here, we established the first transformation protocol for *C. suis*. Due to the low transformation efficiency of most systems (24), we introduced a new parameter as an indicator for transformation efficiency, which is based on the number of transformants at the third passage post-initial infection. This parameter was then used for protocol optimization. We found that CaCl_2_ concentrations over 100 mM significantly increased the number of obtained transformants compared to 50 mM, a concentration used in most published protocols to date (19 – 22), with few exceptions (36). The impact of different antibiotic selections on the transformation outcome has long been described (37) and is reflected here with ampicillin being more effective for *C. suis* transformation than chloramphenicol. As shown for *C. pneumoniae* (21), we confirm that not all strains are equally transformable, with laboratory strain S45 yielding more transformants than field strain SWA-141. Moreover, the larger surface area and the removal of surface proteins following trypsin treatment (38) may facilitate delivery of EBs (39) and DNA into the cell, thus increasing the number of transformants following initial infection of trypsinized cells compared to seeded cells. Next, successful allele-replacement mutagenesis with vector pUC-*trp*GFPinter-mC demonstrates that stable *Chlamydia* transformation is possible using vectors that only possess a pUC *ori*, selection markers, and homology arms. Our observations made for allele replacement of *trpA* with RSGFPCAT cassette-interrupted *trpA* were comparable to findings described for FRAEM (16). For FRAEM, the replication factor *pgp6*/CDS8 of the chlamydial plasmid is put under a Tet promoter, thus allowing the expulsion of the chlamydial plasmid by removal of tetracycline (16). Both vectors allowed the retention of the native plasmid, a recognized virulence factor for *Chlamydia* (40). In contrast, conventional shuttle vectors comprising the whole, unmodified structure of the native chlamydial plasmid usually replace the native plasmid (19). Compared to the FRAEM suicide vector, the massive size reduction from 18 kbp (16) to 10 kbp for pUC-*trp*GFPinter-mC allows greater flexibility in vector design for future studies of the tryptophan synthesis pathway of *Chlamydia*.

The vector pUC-*trp*GFPinter-mC could also be transformed into *C. trachomatis*, though allelic replacement was not observed. Cross-species transformation of chlamydial vectors has only been shown for few *Chlamydia* to date, as species specificity appears to be dependent on the structure of the chlamydial *ori* (23) and plasmid gene *pgp8*/CDS2 (21, 41). It is currently unclear how *C. trachomatis* could maintain the vector over 13 passages in selective antibiotics. This finding will be the subject of future investigations. In contrast, considering the low homology (79%) between the two species in the *trp* region, it was not surprising that no allelic exchange was observed. To date, the exact requirements for interspecies recombination between *Chlamydia* are insufficiently understood. Most events of interspecies recombination have so far been observed following *in vitro* co-infection of species that appear to infect the same inclusion (42), which can be used for the production of hybrid strain libraries (43). For *C. trachomatis*, we now show that transformation of *C. suis* allele-replacement vectors is possible, though the exact extent and requirements are unknown. This is cause for concern considering that the Tet-island is present in many *C. suis* strains (5), that both species have been detected in the eyes of trachoma patients (44), and that first-line antibiotics for treatment of chlamydial infections in humans are azithromycin and doxycycline (45). Unsuccessful transformation attempts of *C. muridarum* indicate that there are interspecies barriers for transformation of allele-replacement vectors similar to what has been identified for conventional shuttle vectors (41). Alternatively, the transformation protocols for *C. trachomatis* and *C. muridarum* were not optimized in this study; therefore, a lack of transformation success may be explained by suboptimal experimental conditions.

Next, we further looked into the *rrn-nqrF* plasticity zone of *C. suis*, which contains the *inv* gene as well as, if present, the Tet-island. This gene, also termed *intimin* or *ilp (invasin*-like protein), only exists in *C. caviae*, *C. suis* and, albeit truncated, in *C. muridarum*, and is part of the chlamydial *rrn-nqrF* plasticity zone (11, 46). In other bacteria such as *Yersinia pseudotuberculosis* and *E. coli*, *inv* is associated with attachment to (*Y. pseudotuberculosis*) or invasion of (*E. coli*) eukaryotic cells. However no distinct function could be identified in *C. suis* (46). If present, the Tet-island divides the 4 kbp *inv* gene of *C. suis* into a 1.5 and 2.5 kbp sequence (5, 7). Whole-genome analysis of multiple *C. suis* field strains has shown that the *inv* gene is likely inactive even if no Tet-island was present (5). Transformation attempts of *C. muridarum* and *C. trachomatis* with *inv* vector pUC-*inv*GFPinter-mC remained unsuccessful, which was expected considering the low homology between the *rrn-nqrF* region of *C. suis, C. trachomatis* and *C. muridarum* (11).

With the successful transformation of *trp* and *inv* vectors, we show that the presence of *C. suis* plasmid sequences is not necessary for the construction of allele-replacement and integration vectors. It is currently unclear why similar vector constructs targeting different chromosomal regions resulted in allelic exchange and complete vector integration for *trp* and *inv* vectors, respectively. Most routinely used vectors to date lead to allelic exchange with loss of the free plasmid vector over time (16, 23). Full integration of a vector into the chromosome has so far been observed for a broad-spectrum vector isolated from *Bordetella bronchiseptica* that was modified for transformation into *C. trachomatis* L2 (35).

Unfortunately, likely due to an overall lower transformation efficiency in field strains, the *inv* vector could not be transformed into the tetracycline-resistant strain SWA-141. It would have been interesting to explore whether the RSGFPCAT cassette would have replaced the Tet-island by allelic exchange, or whether the entire 10 kbp vector would have integrated into the *rrn-nqrF* plasticity zone expanding the region even further. In the case of the laboratory strain S45 RIF, the complete *inv* vector *pUC-inv*GFPinter-mC integrated downstream of *inv* and upstream of *nqrF*, leaving the target gene intact. This finding supports the hypothesis that the *rrn-nqrF* region is a plasticity zone with non-essential genes that might serve as virulence factors (11, 46), and could be a target for future HGT events.

We then modified pUC-*inv*GFPinter-mC to further explore integration dynamics of the *rrn-nqrF* region, including determining the required size of the homology arms for successful integration. Here, we could transform vectors with homology arms as short as 1.5 kbp but not 0.5 kbp. Most chlamydial vectors described to date have homologous sequences ranging between 1.5 and 3 kbp on one or both ends of the cassette (16, 23, 35). In contrast, transient transformation has been reported with shorter homologous sequences using either TargeTron (17) or a transposon mutagenesis system (47, 48). Overall, it is well known that an increasing vector size decreases the transformation efficiency (49). However, the smallest vector used, pUC-*inv*up-mC (4.5 kbp), had a notably low number of transformants at the third passage, which in turn may be explained by the reduced length of the homology arm (1.5 kbp). Alternatively, it is possible that the region close to *nqrF* is more prone to integration regardless of the overall length of the homologous sequence. This hypothesis is supported by the finding that the site of integration was immediately upstream of *nqrF* for both pUC-*inv*down-mC, containing only the 2.5 kbp downstream *inv* sequence, and the original *inv* vector. Furthermore, *in vitro* co-culture experiments have identified the *nqrF* gene as a potential target for homologous recombination (26).

Subsequently, we prepared chlamydial and non-chlamydial vectors to explore the capacity of *C. suis* for transformation and subsequent integration. The vectors included an empty *E. coli* vector pUC-mC, the *A. salmonicida* subsp. *salmonicida* vector pRAS3-3432, and two *C. suis* vectors containing the *OfxX-IScs605* region of pRAS-3432, of which one included the *C. suis* plasmid and the other was constructed as an *inv* integration vector. We achieved transformation only with the *inv* vector, while transformation attempts with the *C. suis* plasmid vector remained unsuccessful. However, this finding could be explained by the considerable size of the *C. suis* plasmid vector (16.5 kbp) combined with the use of chloramphenicol for selection, as well as the *A. salmonicida* subsp. *salmonicida* insert sequence. The latter consideration is supported by the observation that *inv* vector pUC-*inv*pRAS3inter-mC yielded very few transformants. The pUC-*inv*pRAS3inter-mC vector integrated upstream of *nqrF*, leaving chromosomal *inv* intact, as was observed for the interrupted *inv* vector pUC-*inv*GFPinter-mC and the vector pUC-*inv*down-mC which contained only the downstream homology arm of *inv*.

Finally, we found that all *C. suis* transformants retained free *trp* and *inv* vector over thirteen passages, which could be re-transformed into competent *E. coli*. This is surprising considering that previous studies have suggested that the chlamydial *ori* and ORF8/*pgp6* are essential for plasmid maintenance, especially once selective pressure is removed (16, 23, 50). Together with the observed integration and subsequent amplification of various *inv* vectors into *C. suis*, these findings could be indicative for looping-out events of integrated vector from the chromosome. The exact dynamics are currently unclear. Future studies are necessary to better understand the interplay of free vector and allele replacement or integration. Considering that *C. suis* and *C. trachomatis* transformants propagated in ampicillin appeared to result in more deletions and multimers than those grown in chloramphenicol, it is likely that the choice of selection and the antibiotic concentration plays a role in this interplay.

These results represent an interesting contribution to our knowledge concerning the origin of the Tet-island. The current hypothesis based on whole-genome analysis (5, 12) and *in vitro* data (26, 51) is that the Tet-island was the result of a single HGT event where a pRAS3-like plasmid was taken up by natural transformation (3). Following uptake, it has been suggested that the transposase *IScs605*, detected on many Tet-islands and on pRAS3-3432 (7, 9), was responsible for integration into *C. suis* with *inv* being a random target location due to its size and apparent non-functionality (46, 52). The spread of the Tet-island among the *C. suis* then likely occurred via homologous recombination (5, 12, 26). In this study, we show that the transposase appears to have little impact on the site of integration in *C. suis* even after successful transformation and does not aid in the uptake of non-chlamydial DNA. The exact function of *IScs605* in *C. suis* but also other bacterial species such as *A. salmonicida* subsp. *salmonicida* needs to be investigated in future studies. Here, we further confirm genomic findings that the chlamydial *rrn-nqrF* region, and the *C. suis* genome in general, is highly plastic (5, 11).

There are a few limitations of this study. Due to asynchronous growth dynamics of *Chlamydia* (53), and an incomplete understanding of how transformation may initially impact multiplication of transformed *Chlamydia*, it is not optimal to use the third passage as an indicator for transformation efficiency. Better methods to determine the transformation efficiency in *Chlamydia* will have to be developed in the future. Furthermore, although clonal purification is a crucial step for the use of transformed bacteria in experiments (15), we omitted this step for initial confirmation because plaque formation is not visible for *C. suis* and limiting dilution is very challenging with incomplete success (26, 51). To ensure a mostly clonal population of transformants for sequencing, we reduced the number of untransformed *Chlamydia* by growing transformed cultures multiple passages in the presence of antibiotic selection prior to sample collection. While this step does not exclude the possibility of more than one transformed population in the same culture, the low number of transformants obtained at the third passage combined with the above-described stock procedure likely yielded mostly clonal cultures for *trp* and *inv* transformants. Moreover, secondary changes, such as integration, looping-out events, or the loss of free vector may also occur in clonal cultures.

In future studies, we would like to investigate the *rrn-nqrF* plasticity zone in further detail, particularly if complete vector integration is limited to this region and the role of individual genes/operons such as the *rrn, inv*, and *nqrF* during the integration process. With all the new genetic tools available (14, 15), it would be interesting to investigate whether *rrn-nqrF* is equally transformable in other species. The recently published plasmid pBBR1MCS-4 could play a key role in exploring these complex dynamics considering that this broad host-range vector from *Bordetella pertussis* yielded similar results with complete vector integration in the *incA* region, located outside of the *rrn-nqrF* zone, and retention of free vector in the transformants (35). That study further found indication for both complete integration and allelic exchange in multiple regions of interest revealing an even more dynamic picture to be explored in the future.

Finally, we currently have an incomplete understanding of natural transformation in *Chlamydia*. To date, only one competence gene has been recognized, CT336 in *C. trachomatis*, which encodes for a *Bacillus* ComEC homolog (48). However, the rest of the machinery is unclear (3). It is therefore crucial to further investigate the HGT capabilities of *Chlamydia* considering that a single HGT event in *C. suis* led to the spread of an antibiotic resistance-conferring island within the worldwide *C. suis* population (54 – 57). To date, the Tet-island has not yet been detected in humans infected with this potentially zoonotic species, but it must be considered as a potential risk (44, 58, 59).

In summary, this study establishes the first transformation protocol for *C. suis* with subsequent optimization. Furthermore, we established an allele-replacement vector targeting *trp* and various *inv* vectors that resulted in complete integration into the *rrn-nqrF* plasticity zone. The presence of the chlamydial plasmid is not required for *C. suis* transformation but may contribute to higher transformation rates, while homology arms should be at least 1–2.5 kbp. Our findings show an impressive plasticity and transformability of the *C. suis* genome, with the potential for interspecies DNA transfer, especially within the *rrn-nqrF* plasticity zone, which could become an enticing site for future genetic studies not only for *C. suis* but the entire *Chlamydia* genus.

## MATERIALS AND METHODS

### Cell culture and *Chlamydia* strains

All experiments were performed in LLC-MK2 cells derived from Rhesus monkey kidney cells (kindly provided by IZSLER Brescia, Italy), grown at 37°C and 5% CO_2_. Growth and infection medium were prepared as described (28) with minor changes. Briefly, growth medium was used for cell maintenance and seeding, consisting of minimal essential medium with Earle’s salts, 25 mM HEPES, without L-Glutamine (GIBCO, Invitrogen, Carlsbad, CA, USA) supplemented with 10% fetal calf serum, 2 mM GlutaMAX (200 mM, GIBCO), and 0.4 g D-(+)-glucose (Sigma-Aldrich, St. Louis, MO, USA). Infection medium, used for propagation of *C. suis* and all transformation experiments, was growth medium supplemented with 2 g instead of 0.4 g glucose and, if applicable, contained 1.5 µg/mL cycloheximide (Sigma-Aldrich). *Chlamydia* stocks were stored in sucrose phosphate glutamate (SPG) buffer comprising 218 mM sucrose (Sigma-Aldrich), 3.76 mM KH_2_PO_4_ (Sigma-Aldrich), 7.1 mM K_2_HPO_4_ (Merck Eurolab AG, Dietlikon, Switzerland), and 5 mM GlutaMAX (GIBCO) (60).

All chlamydial strains used in this study are listed in Table 1. Semi-purified stocks were prepared as described previously (26). Briefly, strains were grown in LLC-MK2 cells for up to five passages for a final infection rate of 75–100% in T75 flasks (TPP, Trasadingen, Schweiz). Infected cells were then scraped into the supernatant and lysed with mechanical disruption by vigorously vortexing the chlamydial suspension at maximum speed 1 min with sterile 5 mm Ø glass beads (Sigma-Aldrich). Following removal of cellular debris by centrifugation (500 × *g*, 4°C, 10 min), chlamydiae were pelleted (10,000 × *g*, 4°C, 45 min), resuspended in SPG aiming for an approximate stock concentration of 10^9^ to 10^10^ IFU/mL, and stored as aliquots at −80°C.

The exact stock concentration for each stock was determined with titration by sub-passage as described (26, 61), with modifications: SPG stock was inoculated in duplicate onto the first monolayer of a 96-well plate (TPP) and then serially diluted (threefold). After an incubation period of 48 h, monolayers were fixed in methanol (−20°C, 10 min) and an immunofluorescence assay (IFA) was used to visualize the inclusions as described (61). The IFA protocol consisted of 30 min of blocking solution, 1% bovine serum albumin (Sigma-Aldrich) in phosphate-buffered saline (PBS), followed by 1 h incubation with a *Chlamydia*-specific primary antibody (1:200 dilution, *Chlamydiaceae* lipopolysaccharide LPS, Clone ACI-P; Progen, Heidelberg, Germany). Finally, plates were washed with PBS (Gibco) and DNA was stained with 1 µg/mL 4′, 6-diamidino-2′-phenylindole dihydrochloride (DAPI; Molecular Probes, Eugene, OR, USA) and inclusions visualized with a 1:500 diluted Alexa Fluor 488-conjugated secondary goat anti-mouse antibody (Molecular Probes) for 45 min (61). After a final wash with PBS, the infectivity of the stock was expressed as IFU/mL by counting the number of inclusions in a whole well using the 20× objective of a Nikon Ti Eclipse epifluorescence microscope (Nikon, Tokyo, Japan).

### Vector construction

Primers, plasmids, and targeted regions used for construction are listed in Table S2 for each vector. DNA from strain S45 was used for all *C. suis*-derived vector regions (Table 1). The pUC regions comprising pUC origins of replication (*ori*), beta-lactamase gene (*bla*) and either mCherry (*mC*) or a fused GFP/chloramphenicol resistance gene (RSGFPCAT), with corresponding promoters, were amplified from pBOMB4R-MCI (kindly provided by Ted Hackstadt, MT, USA) (33) and pGFP::SW2 (kindly provided by Ian N. Clarke, Southampton, UK) (19), respectively. The pRAS3-3432 plasmid was obtained from *A. salmonicida* subsp. *salmonicida* strain SHY16-3432 (9). PCR was performed with either Phusion Hot Start II (Thermo Fisher Scientific, Waltham, MA, USA) or LongAmp Taq (New England Biolabs, NEB, Ipswich, MA, USA) DNA Polymerase according to the manufacturer’s instructions (Table S2). Elongation time was 60 s and 50 s per kbp target region for Phusion and LongAmp polymerase, respectively. PCR products were run on a gel according to standard molecular methods using 1% agarose. Resulting amplicons with the correct size were cut out of the gel and purified with the GeneJet Gel Extraction Kit (Thermo Fisher). The HiFi DNA Assembly Cloning Kit (NEB), as recommended by the manufacturer, assembled the backbone (containing pUC), and inserted the region of choice. All vectors used in this study are listed in Table 2.

### Cloning and vector preparation

Assembled constructs were cloned into NEB5-alpha (<12 kbp vectors) or NEB10-beta (>12 kbp vectors) competent *E. coli* (NEB) according to the manufacturer’s instructions. Selective antibiotics included ampicillin (Thermo Fisher, 100 mg/mL in ddH_2_O) and/or chloramphenicol (Sigma-Aldrich, 50 mg/mL in ethanol) or tetracycline (Sigma-Aldrich, 10 mg/mL in ddH_2_O) depending on the resistance markers of the construct (Table 2), at working concentration of 100 µg/mL (*bla*), 25 µg/mL (*cat*), and 10 µg/mL (*tet*), respectively. Clones were confirmed by Sanger sequencing (Microsynth, Balgach, Switzerland). Unmethylated plasmid DNA was generated in dam−/dcm− strain (NEB) prior to chlamydial transformation (23). Plasmid DNA was extracted with GeneJet Maxiprep (Thermo Fisher) and concentration was measured with a Nanodrop 2000c spectrophotometer (Thermo Fisher).

### Chlamydial transformation

Transformation experiments comprised the following steps per condition: 7.5 µg vector DNA was mixed with 2.5 × 10^6^ to 1.25 × 10^7^ IFU of chlamydial SPG stock in 100 µL of CaCl_2_ comprising 20 mM Tris (Roche, Basel, Switzerland) and 100 mM CaCl_2_ (Merck), adjusted to a pH of 7.4. The mixture was then incubated at room temperature for 1 h with mild agitation as described (21). In the meantime, LLC-MK2 cells were trypsinized, and approximately 2.5 × 10^6^ cells were mixed with infection medium without cycloheximide and pelleted (500 g, 5 min). After pelleting the cells, the supernatant was replaced with 150 µL of 100 mM CaCl_2_, mixed gently with the vector/*Chlamydia* suspension and immediately added to the cultivation medium without cycloheximide to avoid cell death, aiming for a final suspension volume of 6 mL. We then plated the suspension onto three wells of a six-well plate (2 mL/well) and let the cells settle on an even surface at room temperature for approximately 30 min. Next, centrifugation (1000 × *g*, 1 h, 35°C) was performed and cultures were incubated at 37°C and 5% CO_2_. After 6 h of incubation, cycloheximide (1.5 µg/ml) was added to each well, as well as either ampicillin (5 µg/ml), chloramphenicol (0.5 µg/mL) or tetracycline (0.06 µg/mL), depending on the vector used and the protocol. At 36 hpi, the first passage was performed by removing the supernatant, mixing vigorously, and returning 0.5 mL of supernatant back to each well. Cells were then scraped into the supernatant, mechanically disrupted with glass beads (3 mm Ø, Sigma-Aldrich) and pelleted at 500 g (5 min, 35°C). Next, the *Chlamydia*-containing supernatant was added to well 1 of a fresh six-well plate seeded to confluence with LLC-MK2 cells, and a serial dilution (1:4) was performed into two more wells. Following centrifugation (1000 × *g*, 1 h, 35°C), inocula were replaced with infection medium containing cycloheximide and either 5 µg/mL ampicillin, 0.5 µg/mL chloramphenicol, or 0.125 µg/mL tetracycline, depending on the experiment. Cultures were passaged up to four times. Passage 3 was used to determine transformant progeny (see below), while Passage 4 was used for qualitative assessment of successful (visible transformants) and unsuccessful (no transformants) transformation. Individual transformants used for stability assay and whole-genome sequencing were further processed by passaging them six times at 25 µg/mL ampicillin, 1 µg/mL chloramphenicol, or 1 µg/mL tetracycline. At Passage 6, three wells of a six-well plate per transformant were infected and collected at 48 hpi as described for stock preparation. Transformants with vectors containing two different antibiotic resistance genes were processed separately in both antibiotics.

### Transformant progeny at Passage 3

At the third passage (Passage 3), only 1 mL of suspension was used for inoculation of the first well. Serial dilution was performed as described for transformation. Depending on the number of visible transformants, inocula from well 3 (many visible transformants) or well 2 (none or few transformants) were used to infect four LLC-MK2 monolayers on glass coverslips (13 mmØ, Thermo Scientific) in a 24-well plate (TPP). Following centrifugation (1000 × *g*, 1 h, 35°C), we replaced the inocula with fresh infection medium supplemented with cycloheximide and the antibiotic selection of choice. After 24–48 h (*C. suis, C. trachomatis*: 48 h; *C. muridarum*: 24 h), monolayers were fixed in methanol and stained with 1 µg/mL DAPI in PBS for 5 min. Finally, coverslips were mounted with FluoreGuard mounting medium (Hard Set; ScyTek Laboratories Inc., Logan, UT, USA) on glass slides and the number of transformed inclusions was counted on the whole coverslips or in 30 ocular grids (area: 0.2775 mm^2^) using the 20× objective (PL FLUOTAR 20×/0.50 PH 2, ‘/0.17/B) of a Leica DMLB fluorescence microscope. The number of transformants was then expressed as transformant progeny at Passage 3 in IFU/mL.

Each transformation experiment included a control shuttle vector as a parallel reaction containing either the pGFP::SW2 (19) or pBOMB4R-MCI (33) backbone fused with the species-specific chlamydial plasmid. This control allowed a quantitative determination of the overall number of transformants at Passage 3 and was used as an indicator for the validity of individual transformation experiments. *C. suis* control vectors were named pUC-Cspl-GFP and pUC-Cspl-mC, and the *C. muridarum* vector was named pUC-Cmpl-mC. For *C. trachomatis,* pBOMB4R-MCI was used.

### Stability assay

We passaged each transformant of interest three times in the presence or absence of the selective antibiotic used for stock preparation by infecting fresh monolayers in 24-well plates as described above. Following centrifugation, inocula of the four infected wells were replaced with infection medium containing cycloheximide. Selective antibiotics were added to two of the four wells for culture in the presence and absence of selection. All passages were fixed in methanol 48 hpi, stained with DAPI as described for transformant progeny determination and mounted onto glass slides. The amount of transformed versus untransformed inclusions was qualitatively assessed at 20× and under oil immersion at 1,000× magnification with a 100× objective (PL FLUOTAR 100×/1.30, OIL, ‘/0.17/D, Leica Microsystems) using the Leica fluorescence microscope. Representative images were taken for documentation.

### Whole-genome sequencing and analysis

Genomic DNA was extracted using the MasterPure Complete DNA and RNA Purification Kit (Lucigen, LGC Biosearch Technologies, Teddington, UK). Multiplex libraries were prepared using the SQK-LSK112 ligation sequencing kit with EXP-NBD112.24 barcodes and sequenced on an R10.4 flow cell (Oxford Nanopore Technologies, Oxford, UK). Basecalling (high accuracy), adapter trimming, and demultiplexing were performed with Guppy v6.1.1 (Oxford Nanopore Technologies). Host sequences were removed by mapping reads to the *Macaca mulatta* genome (GCF_003339765.1) and collecting unmapped reads using minimap2 v2.24 (62) and samtools 1.15.1 (63). Following read filtering to a minimum length of 1,000 bp with nanoq v0.9.0 (64), assemblies were generated with Flye v2.9.1 (65) and polished with Racon 1.5.0 (66) and Medaka v1.7.2 (66). Annotations were performed with Bakta 1.6.1 (67). Regions of interest were extracted in CLC Main Workbench 22 and compared with Easyfig 2.2.5 (34). Sequence alignments and similarity calculations were performed using Clustal Omega (68).

### Statistical analysis

GraphPad Prism (v.9, GraphPad Software, Boston, MA, USA) was used for all statistical analyses using either the one-way analysis of variance (Kruskal-Wallis test) with Dunn’s multiple comparisons test for multiple means, or the Mann-Whitney test for the comparison of two means. A *P* value ≤0.05 was considered significant unless otherwise mentioned in the text.

## Data Availability

Genome assemblies are available under NCBI Bioproject no. PRJNA962280.

## SUPPLEMENTAL MATERIAL

Supplementary Figures spectrum.02378-23-s0001.pdf This is a merged PDF with all supplementary figures (Figure S1–S5).

Table S1 spectrum.02378-23-s0002.xlsx Shown is the sequencing data of *Chlamydia suis* and *Chlamydia trachomatis* transformants.

Table S2 spectrum.02378-23-s0003.xlsx Vector construction. Shown are all vectors used in this study either for transformation or construction of more complex vectors.

ASM does not own the copyrights to Supplemental Material that may be linked to, or accessed through, an article. The authors have granted ASM a non-exclusive, world-wide license to publish the Supplemental Material files. Please contact the corresponding author directly for reuse.
