# Supplementary material for: Chlamydia suis displays high transformation capacity with complete cloning vector integration into the chromosomal rrn-nqrF plasticity zone
Source: Microbiol Spectr. 2023 Oct 26;11(6):e02378-23. doi: 10.1128/spectrum.02378-23 (PMC10715202; doi:10.1128/spectrum.02378-23)
Supplement: Supplementary Figures — This is a merged PDF with all supplementary figures (Figure S1–S5). [file spectrum.02378-23-s0001.pdf]

Figure S1. ***Chlamydia suis*** **transformation: Optimization data**

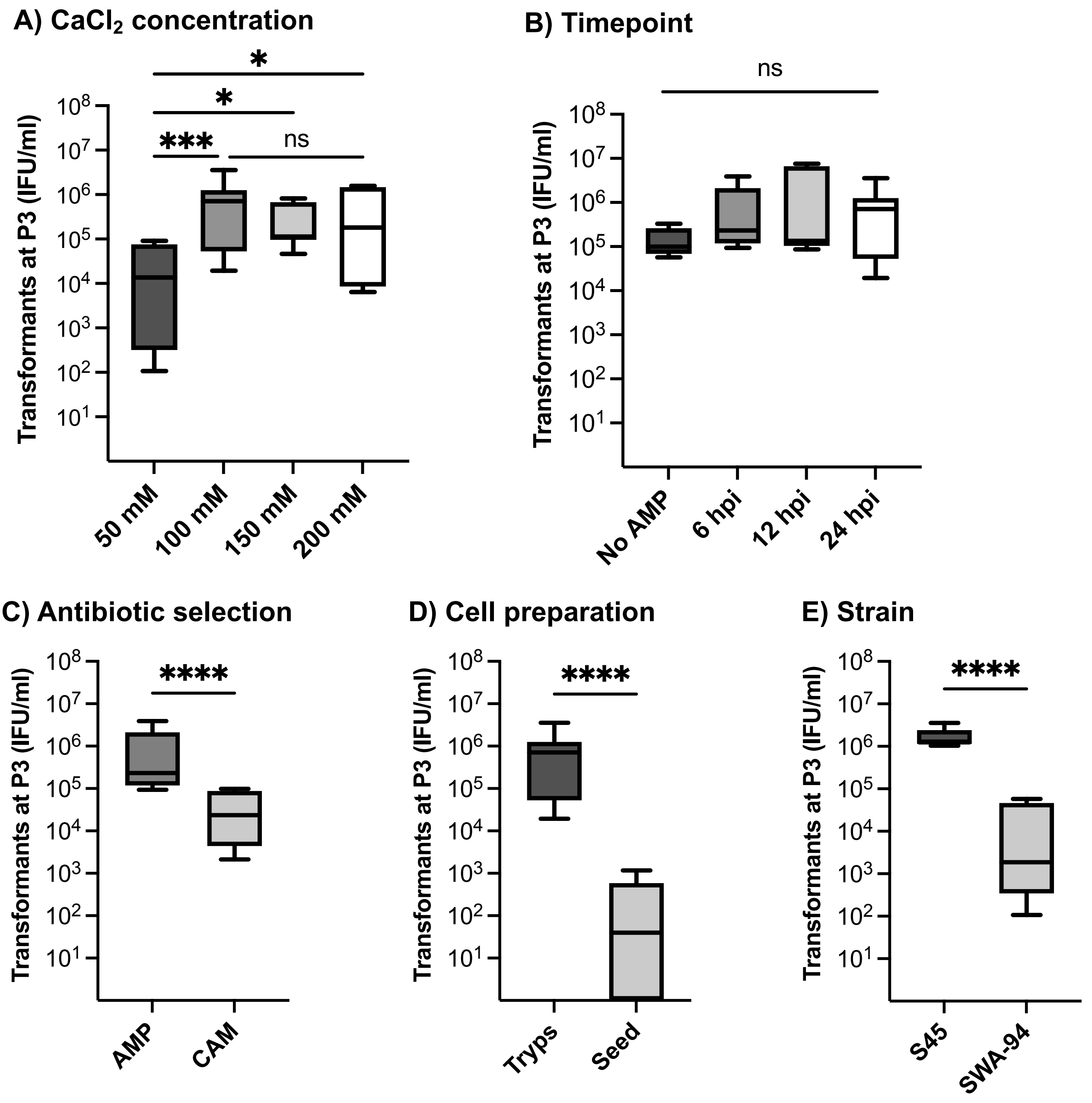

Figure S5. Transformation optimization.

Transformant progeny was determined by the number of transformants at Passage 3, expressed as inclusion forming units per mL (IFU/ml). Conditions analyzed included the effects of **A)** the calcium chloride concentration (50-200 mM) and **B)** the timepoint of initial ampicillin (AMP) selection post initial infection (hours post infection, hpi). Additionally, the transformant progeny was compared between **C)** ampicillin (AMP) and chloramphenicol (CAM) for selection, **D)** the use of trypsinized cells and seeded cells for initial infection, and **E)** between the laboratory strain S45 RIF and field strain SWA-94. Statistical tests were performed with GraphPad Prism 9 following three independent experiments, using the Kruskal-Wallis test with Dunn's multiple comparisons test for multiple comparisons and the Mann-Whitney test for comparing two values. Significant differences were represented with asterisks: Four asterisks (****) represent p-values <0.0001, three (***) represent p-values <0.001 and one (*) represents p-values 0.01-0.05. Non-significant values were labeled with ns.

Figure S2. Transformation of *C. muridarum, C. suis* and *C. trachomatis* with *trp* allele-replacement vector pUC-*trp*GFPinter-mC

A) *Chlamydia muridarum:* Transformation with pUC-*trp*GFPinter-mC

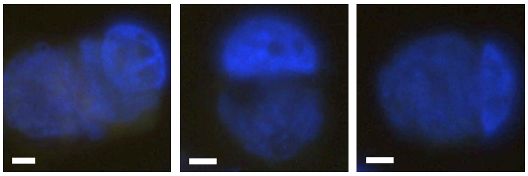

***C. muridarum* could not be transformed with pUC-*trp*GFPinter-mC in three independent experiments.** Shown are representative images of $GFP^{neg}mCherry^{neg}$ *C. muridarum* inclusions after 4 passages in the presence of ampicillin following transformation attempt with pUC-*trp*GFPinter-mC. Images from each channel – DAPI (blue), GFP (green), mCherry (red) – were taken individually and merged. Size bar indicates 5 µm.

B) *Chlamydia suis:* Transformation with pUC-*trp*GFPinter-mC

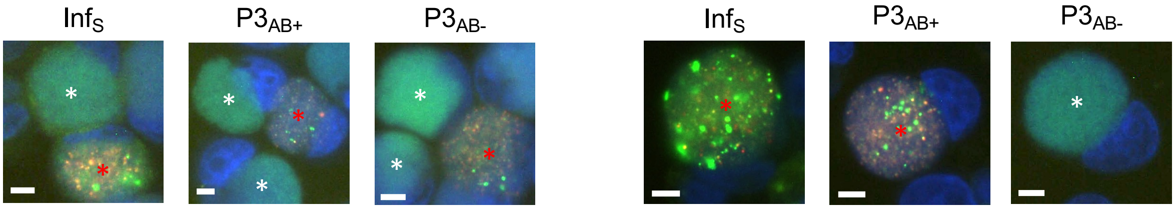

***C. suis* transformants grown in chloramphenicol.** Shown are representative images of *C. suis* cultures (strains S45 RIF) successfully transformed with pUC-*trp*GFPinter-mC. Images from each channel – DAPI (blue), GFP (green), mCherry (red) – were taken individually and merged. The images display *C. suis* transformants that are either $GFP^{pos}mCherry^{pos}$ (red asterisk) or $GFP^{pos}mCherry^{neg}$ (white asterisk). The latter inclusions are indicative for allele-replacement with the chromosomal *trp* region. Samples were grown in chloramphenicol (left) or ampicillin (right). Size bar indicates 5 µm. Inf_S = Start of stability assay, infection in selective antibiotics. $P3_{AB+}$= Stability assay after passage 3 in selective antibiotics. $P3_{AB-}$= Stability assay after passage 3 without antibiotics.

C) *Chlamydia trachomatis:* Transformation with pUC-*trp*GFPinter-mC

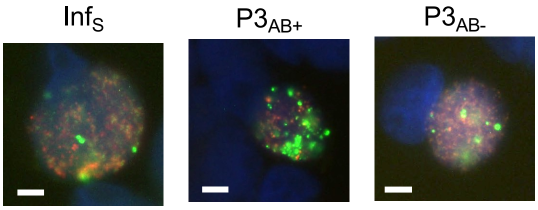

***C. trachomatis* transformants grown in chloramphenicol.** Shown are representative images of pUC-*trp*GFPinter-mC transformants grown in chloramphenicol. Images from each channel – DAPI (blue), GFP (green), mCherry (red) – were taken individually and merged. Size bar indicates 5 µm. Inf_S = Start of stability assay, infection in selective antibiotics. $P3_{AB+}$= Stability assay after passage 3 in selective antibiotics. $P3_{AB-}$= Stability assay after passage 3 without antibiotics.

Figure S3. *Invasin* vectors could not be taken up by *C. trachomatis, C. muridarum* or a tetracycline-resistant *C. suis* field strain

A) *C. trachomatis* and *C. muridarum:* Transformation with pUC-*inv*GFPinter-mC

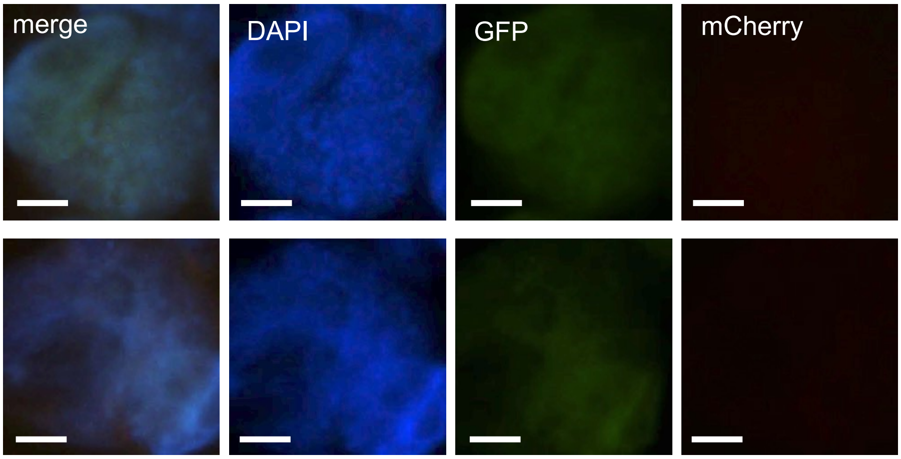

C. trachomatis

C. muridarum

***C. trachomatis and C. muridarum* could not be transformed with pUC-*inv*GFPinter-mC in three independent experiments.** Shown are representative images of GFP^{neg}mCherry^{neg} *C. trachomatis* (top) and *C. muridarum* (bottom) inclusions after 4 passages in the presence of ampicillin following transformation with pUC-*inv*GFPinter-mC. Images from each channel – DAPI (blue), GFP (green), mCherry (red) – were taken individually and merged (left). Individual images are shown for DAPI, GFP and mCherry (left to right). Size bar indicates 5 µm.

B) *Chlamydia suis:* Transformation with pUC-CspI-mC pUC-*inv*GFPinter-mC

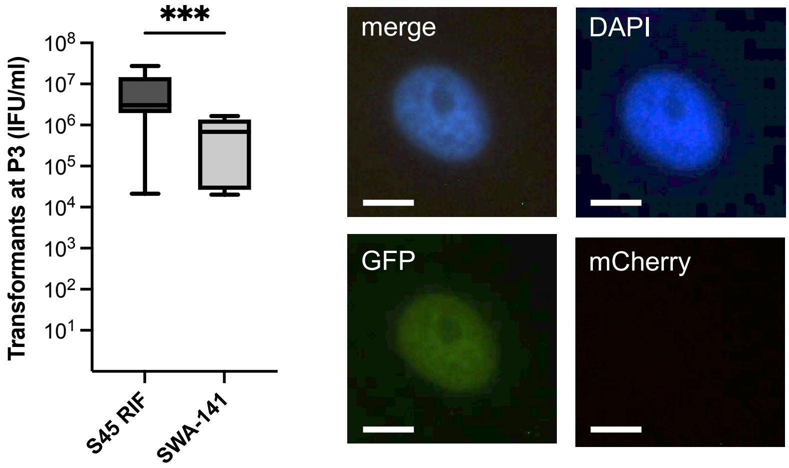

Tetracyline-resistant field strain SWA-141 could not be transformed with pUC-*inv*GFPinter-mC in four independent experiments. The left panel compares the number of transformants of S45 RIF at Passage 3 with that of SWA-141 for control vector pUC-CspI-mC, expressed as IFU/ml. Statistical tests were performed with GraphPad Prism 9 after three to four independent experiments, using the Mann-Whitney test. Significant differences were represented with asterisks: Three (***) represent p-values <0.001. The right panel shows representative images of *C. suis* strain SWA-141 following transformation attempts with pUC-*inv*GFPinter-mC. Images from each channel – DAPI (blue), GFP (green), mCherry (red) – were taken individually and merged. Size bar indicates 5 µm.

Figure S4. Transformation of *C. suis strain* S45 RIF with *Invasin*-based vectors

A) Stability assay of partial *inv* vectors

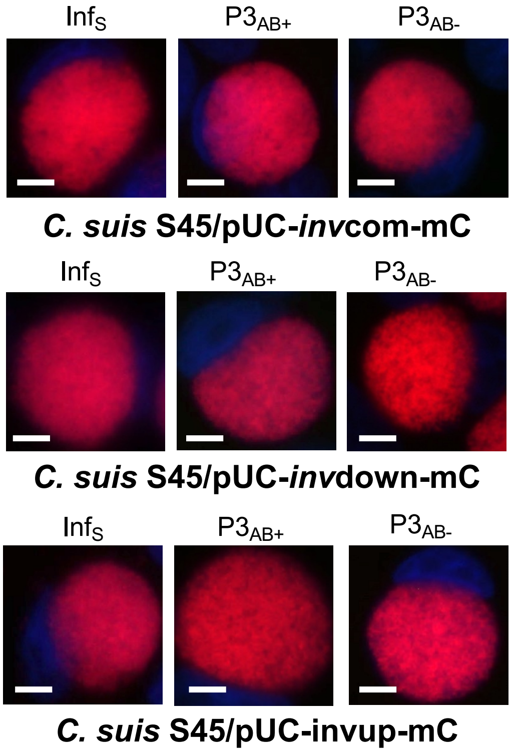

B) Transformation efficiency *inv* vectors

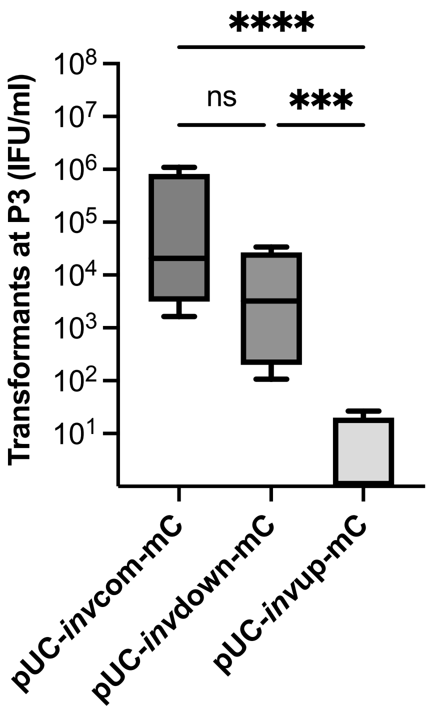

C) Whole-genome sequencing of *C. suis* S45/pUC-*inv*com-mC transformants

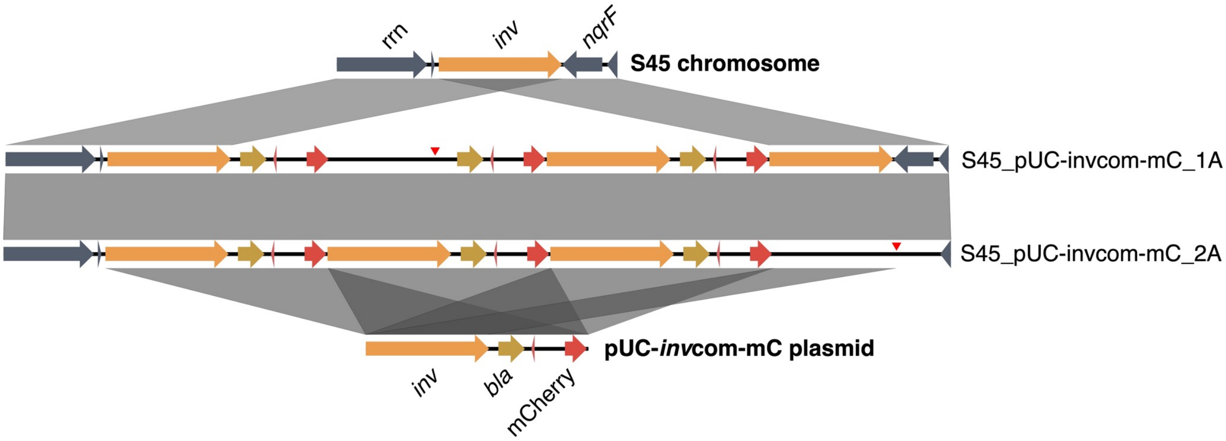

A) Shown are representative images of *C. suis* strain S45 RIF successfully transformed with pUC-*inv*com-mC, pUC-*inv*down-mC and pUC-*inv*up-mC. Images from both channels – DAPI (blue), mCherry (red) – were taken individually and merged. Samples were grown in ampicillin. Size bar indicates 5 µm. Inf$_S$ = Start of stability assay, infection in selective antibiotics. P3$_{AB+}$= Stability assay after passage 3 in selective antibiotics. P3$_{AB-}$= Stability assay after passage 3 without antibiotics. **B)** The number of transformants was determined at Passage 3 and expressed as inclusion forming units per mL (IFU/ml). We compared the transformation efficiency of pUC-*inv*com-mC with pUC-*inv*down-mC and pUC-*inv*up-mC using the Kruskal-Wallis test with Dunn's multiple comparisons. Four asterisks (****) represent p-values <0.0001, three (***) represent p-values <0.001. Non-significant results are indicated with ns. **C)** Sequence comparison of plasmid pUC-*inv*com-mC, the chromosomal *inv* region in native strain S45 (orange), and the corresponding region in two transformants. Grey boxes between sequences indicate homologous regions. Additional chromosomal regions are shown in dark blue; *bla*, and mCherry are shown in dark yellow and red, respectively. Deletions leading to contraction of the ORFs are highlighted with a vertical arrow tip (red). The figure was generated using EasyFig 2.1.

Figure S5. *Invasin* vectors, but not chlamydial plasmid vectors, allow integration of up to 10 kbp non-chlamydial DNA into the *rrn-nqrF* region

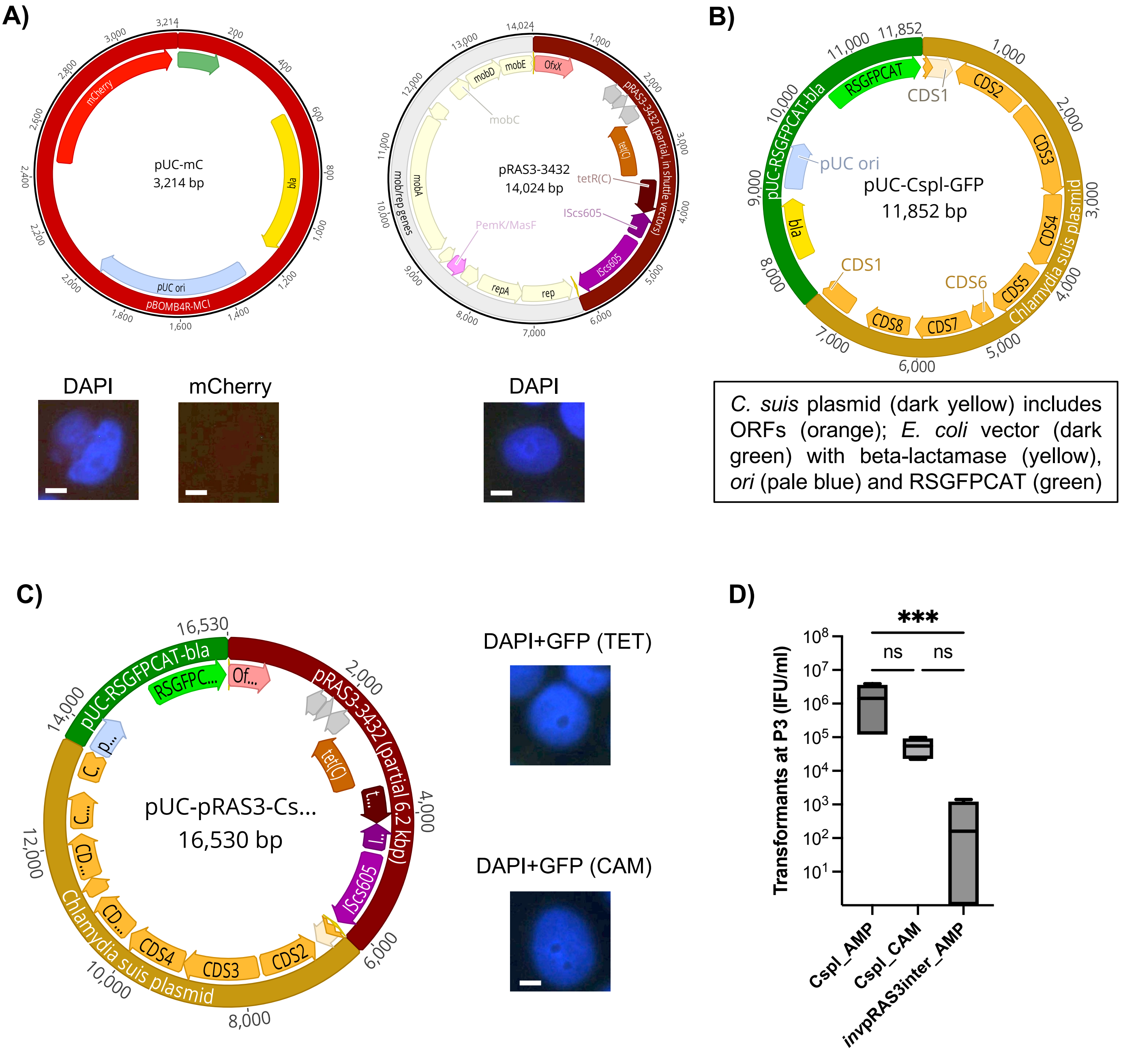

A) Shown are the vector maps of pUC-mC, pRAS3-3432, **B)** pUC-Cspl-GFP and **C)** pUC-pRAS3-Cspl-mC (tetracycline [TET] selection: top; chloramphenicol [CAM]: bottom), as well as representative images of the transformation attempts. For pUC-mC, images from channels DAPI (blue) and mCherry (red) were taken individually and merged. For pRAS3-3432, only DAPI images were taken. For pUC-pRAS3-Cspl-GFPCAT, DAPI (blue) and GFP (green) channel images were taken and merged. Size bar indicates 5 µm. **D)** Number of transformants was counted at Passage 3, expressed as inclusion forming units per mL (IFU/ml). We compared the number of transformants of pUC-Cspl-GFP selected with ampicillin (Cspl_AMP) or chloramphenicol (Cspl_AMP) and pUC-*inv*pRAS3inter-mC selected with ampicillin (*inv*pRAS3inter_AMP) using the Kruskal-Wallis test with Dunn's multiple comparisons. Three asterisks (***) represent p-values <0.001. Non-significant results are indicated with ns.
